# Risk factors for pressure ulcer recurrence following surgical reconstruction: A cross-sectional retrospective analysis

**DOI:** 10.3389/fsurg.2023.970681

**Published:** 2023-03-03

**Authors:** Yueh-Ju Tsai, Cen-Hung Lin, Yuan-Hao Yen, Cheng-Chun Wu, Carolina Carvajal, Nicolas Flores Molte, Pao-Yuan Lin, Ching-Hua Hsieh

**Affiliations:** Department of Plastic Surgery, Kaohsiung Chang Gung Memorial Hospital and Chang Gung University College of Medicine, Kaohsiung, Taiwan

**Keywords:** risk factor, recurrence, pressure ulcer, surgical reconstruction, decision tree model

## Abstract

Many studies on the recurrence of pressure ulcers after surgical reconstruction have focused on surgical techniques and socioeconomic factors. Herein, we aimed to identify the risk factors of the associated comorbidities for pressure ulcer recurrence. We enrolled 147 patients who underwent pressure ulcer reconstruction and were followed up for more than three years. The recurrence of pressure ulcers was defined as recurrent pressure ulcers with stage 3/4 pressure ulcers. We reviewed and analyzed systematic records of medical histories, including sex, age, associated comorbidities such as spinal cord injury (SCI), diabetes mellitus (DM), coronary artery disease, cerebral vascular accident, end-stage renal disease, scoliosis, dementia, Parkinson's disease, psychosis, autoimmune diseases, hip surgery, and locations of the primary pressure ulcer. Patients with recurrent pressure ulcers were younger than those without. Patients with SCI and scoliosis had higher odds, while those with Parkinson's disease had lower odds of recurrence of pressure ulcers than those without these comorbidities. Moreover, the decision tree algorithm identified that SCI, DM, and age < 34 years could be risk factor classifiers for predicting recurrent pressure ulcers. This study demonstrated that age and SCI are the two most important risk factors associated with recurrent pressure ulcers following surgical reconstruction.

## Introduction

The management of pressure ulcers is a significant challenge for healthcare professionals. Despite advances in information and technological progress for prevention, the recurrence of pressure ulcers is not rare (1–7). Many of these recurrent ulcers require prolonged time in wound care and even surgery management, both of which often result in costly procedures, lengthy hospitalizations, expensive dressing changes, and worsened quality of life for these people (8–13). To achieve successful surgical reconstruction, thorough preoperative wound care, patient compliance, control of comorbidities, professional postoperative support, and sufficient pressure relief are essential (14). Krause and Broderick suggested that lifestyle, exercise, and diet are protective mechanisms against the recurrence of pressure ulcers (15). Furthermore, lack of social support, inadequate pressure sore prevention knowledge (2, 5, 16, 17), unemployment, and residing in a nursing home (2, 12) have been considered important issues related to recurrence. Regarding demographic and medical factors, it has been reported that male sex, younger age, and a history of previous pressure sore surgery are associated with the recurrence of pressure ulcers (2, 12). However, as many studies describing recurrence after reconstruction have focused on socioeconomic factors (2, 17, 18), education (18), marital status (19), and surgical techniques (20–26), available data on factors associated with recurrence following surgical repair of pressure ulcers are rather limited.

In this study, we aimed to identify the risk factors associated with comorbidities of pressure ulcer recurrence following surgical reconstruction. In addition, we adopted the decision tree method, which is a machine learning model composed of decision rules based on optimal feature cutoff values that split independent variables into different groups in a hierarchical manner to predict an outcome (27–29), to explore the variables that could be used to identify individuals at risk of pressure ulcer recurrence following surgical reconstruction.

## Materials and methods

This study was approved by the Institutional Review Board (IRB) of Chang Gung Memorial Hospital (approval number 201701802B0). The need for informed consent was waived according to IRB regulations because the study was designed for a retrospective analysis of the registered database. In this study, 147 bed-ridden patients who underwent reconstruction for pressure ulcers from 2007 to 2014 were enrolled and followed up for more than three years. The recurrence of pressure ulcers was defined as recurrent pressure ulcers with stage 3, full-thickness ulcer that might involve the subcutaneous fat, or stage 4, full-thickness ulcer with the involvement of the muscle or bone. The systematic records of medical histories, including sex, age, associated comorbidities such as spinal cord injury (SCI), diabetes mellitus (DM), coronary artery disease (CAD), cerebral vascular accident (CVA), end-stage renal disease (ESRD), scoliosis, dementia, Parkinson's disease, psychosis, autoimmune diseases, hip surgery, and locations of the primary pressure ulcer were reviewed. In this study, primary sacral pressure ulcers were treated with perforator flaps or rotation gluteal flaps, ischial pressure ulcers with muscle flaps (gluteal muscle or biceps femoris muscle) and skin flaps (rotation gluteal flap or posterior thigh flap), and trochanteric pressure ulcers with a gluteal rotation flap or pedicled anterolateral thigh flap.

### Statistical analysis

Statistical analyses were performed using IBM SPSS Statistics for Windows, version 20.0 (IBM Corp., Armonk, NY, USA). Descriptive statistics were obtained by calculating the mean and standard deviation for continuous variables and the relative frequencies for categorical variables. These groups were compared using the chi-squared test for categorical variables with odds ratios (OR) and 95% confidence intervals (CIs). Student's t-test was used for the analysis of continuous variables. Statistical significance was set at *p* value < 0.05.

### Decision tree classifier

The decision tree classification model was established by classification and regression tree (CART) analysis (30, 31) using the rpart function in the rpart package in R based on the Gini impurity index. CART analysis was used to search for the split on each variable to partition the data into two groups: one group of mostly “1s” (people who had sustained recurrent pressure ulcers) and another group of mostly “0s” (people who did not have recurrent pressure ulcers). The CART model identified the best overall split by iteratively testing all possible splits and creating a specified number of nodes until a further reduction in node impurity became impossible or the specified stopping criteria were reached (32–34). In this study, the complexity parameter (*α*) of the “cost-complexity” pruning method is set to 0.001. The complexity parameter (*α*) indicated a measure of how much additional accuracy a split must add to the entire tree to warrant additional complexity. A confusion matrix was used to determine the performance of the decision tree model for the presence of recurrent pressure ulcers. Accuracy, sensitivity, specificity, and area under the curve (AUC) of the receiver operating characteristic (ROC) curve were measured.

## Results

Of the enrolled 147 patients, 46 had recurrent pressure ulcers. As shown in Table 1, among these patients, SCI was the most common associated comorbidity (*n* = 31, 67.4%), followed by DM (*n* = 21, 45.7%), CVA (*n* = 8, 17.4%), and scoliosis (*n* = 8, 17.4%). Among the patients without recurrence, DM was the most common associated comorbidity (*n* = 39, 38.9%), followed by CVA (*n* = 30, 30.0%), SCI (*n* = 24, 23.8%), and Parkinson's disease (*n* = 16, 15.8%). Patients with recurrent pressure ulcers were significantly younger than those without recurrence (55.7 ± 17.4 vs. 62.8 ± 17.4, respectively, *p* = 0.023). Patients who had SCI and scoliosis had significantly higher odds of recurrence of pressure ulcers than those without (SCI, OR = 6.63, 95% CI = 3.08–14.29; scoliosis, OR = 10.42, 95% CI = 2.12–51.31). In contrast, those patients who had Parkinson's disease had significantly lower odds of recurrence of pressure ulcers than those without (OR = 0.12, 95% CI = 0.02–0.92). There were no significant differences in sex, location of ulcer, and associated comorbidities, such as DM, CAD, CVA, ESRD, hip surgery, dementia, psychosis, and autoimmune diseases.

**Table 1:** Characteristics of patients with and without recurrence of pressure ulcer following reconstruction.

	Recurrence (*n* = 46)	No recurrence (*n* = 101)		
			OR	95%CI
Male, *n* (%)	29 (63.0%)	50 (49.5%)	1.27	0.89–2.43
Spine cord injury (SCI), *n* (%)	31 (67.4%)	24 (23.8%)	6.63*	3.08–14.29
Diabetes mellitus (DM), *n* (%)	21 (45.7%)	39 (38.9%)	1.34	0.66–2.70
Coronary artery disease (CAD), *n* (%)	4 (8.7%)	13 (12.9%)	0.65	0.20–2.10
Cerebral vascular accident (CVA), *n* (%)	8 (17.4%)	30 (30.0%)	0.49	0.20–1.16
End-stage renal disease (ESRD), *n* (%)	1 (2.2%)	11 (11.0%)	0.18	0.02–1.45
Scoliosis, *n* (%)	8 (17.4%)	2 (2.0%)	10.42*	2.12–51.31
Hip surgery, *n* (%)	3 (6.5%)	7 (7.0%)	0.94	0.23–3.80
Dementia, *n* (%)	4 (8.7%)	8 (8.0%)	1.11	0.32–3.88
Parkinson's disease, *n* (%)	1 (2.2%)	16 (15.8%)	0.12	0.02–0.92
Psychosis, *n* (%)	1 (2.2%)	1 (1.0%)	2.22	0.14–36.33
Autoimmune diseases, *n* (%)	1 (2.2%)	3 (3.0%)	0.73	0.07–7.17
Locations				
Ischium, *n* (%)	27 (58.7%)	22 (21.8%)	5.1	2.4–10.8
Hip, *n* (%)	6 (13.0%)	11 (10.9%)	1.2	0.42–3.6
Sacrum, *n* (%)	13 (28.3%)	64 (63.4%)	0.22	0.10–0.47
Others, *n* (%)	0 (0.0%)	4 (4.0%)		

Classification by Decision Tree Algorithm. *Marks the significantly higher odds in all factors.

**Figure 1:**
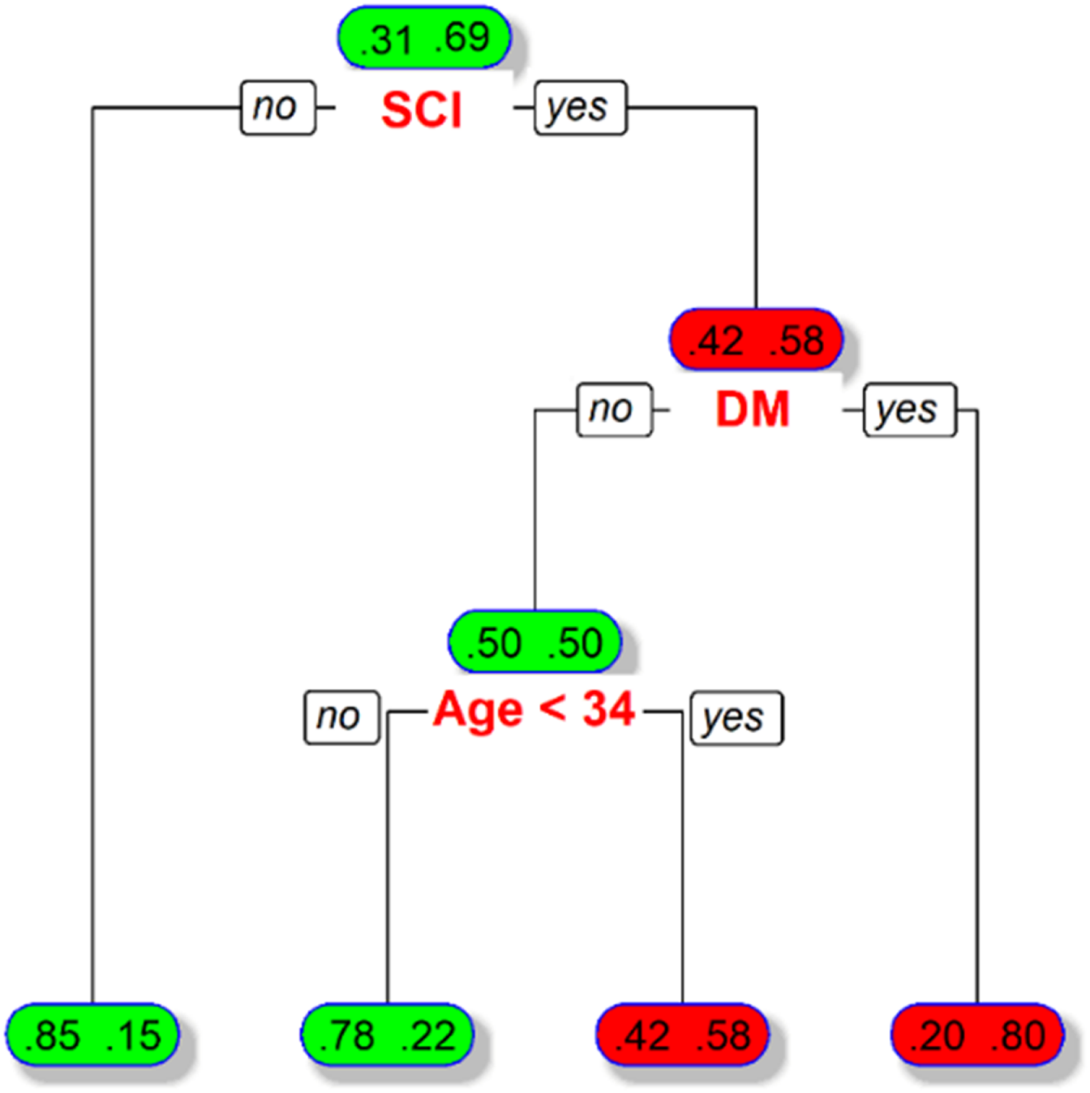
Illustration of decision tree model for predicting recurrence of pressure ulcers in the patients receiving reconstruction for pressure ulcer. Boxes denote the percentage of patients with discriminating variables from CART analysis. Patients with and without recurrence of pressure ulcers are indicated by the fractional number inside the right and left sides of the boxes, respectively.

**Figure 2:**
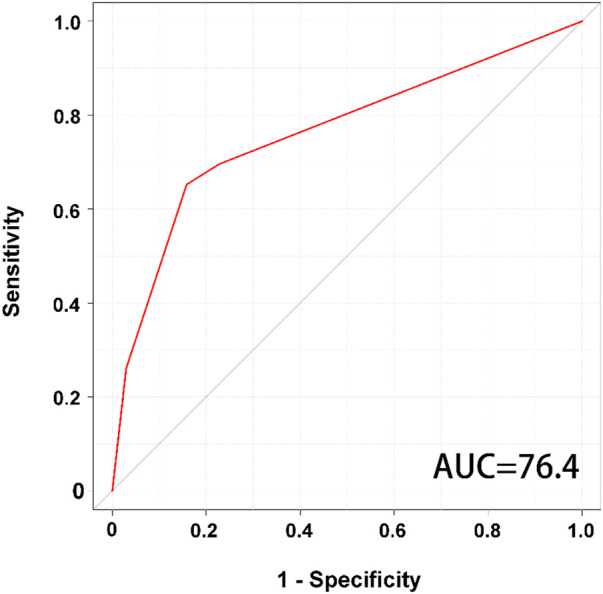
Illustration of AUCROC curves for the decision tree model.

According to the classification by the decision tree algorithm, three groups of patient characteristics (SCI, DM, age < 34 years) with a high risk of recurrent pressure ulcers were identified (Figure 1). The presence or absence of SCI in the DT model was identified as a variable for the initial split. Among patients with SCI, 69% had recurrent pressure ulcers and 31% did not. Among patients with SCI, the presence or absence of DM was identified as a variable in the second split. For this node, 80% of patients with DM had recurrent pressure ulcers. An age of less than 34 years served as the third split for patients without DM. For this node, 58% of patients aged < 34 years had recurrent pressure ulcers. With all variables in the model, the decision tree algorithm achieved an accuracy of 78.23% (sensitivity of 65.22% and specificity of 84.16%). The decision tree model had an AUC of 0.764 for predicting the recurrence of pressure ulcers (Figure 2).

## Discussion

Authors should discuss the results and their interpretations from the perspective of previous studies and working hypotheses. The findings and their implications should be discussed in the broadest context possible. Future research directions may also be highlighted. This study demonstrated that patients with recurrent pressure ulcers were significantly younger and had a higher rate of sustaining SCI and scoliosis but a lower rate of Parkinson's disease than patients without recurrence. In addition, the decision tree algorithm identified that SCI, DM, and age < 34 years could be used as risk factor classifiers for predicting recurrent pressure ulcers.

This study recognized SCI as a risk factor, either from conventional comparisons or decision tree algorithms. Patients with SCI had a 6.6 times higher risk of pressure ulcer recurrence following surgical reconstruction. SCI has been recognized as a major risk factor for the recurrence of pressure ulcers (35, 36), and the recurrence rate can even be as high as 48 to 56% among these patients (5, 37). Furthermore, although older age might be responsible for delayed wound healing and was suggested to be a risk factor for the occurrence of pressure ulcers, this study recognized that younger age is a risk factor for the recurrence of pressure ulcers following surgical reconstruction. This is in accordance with the observation from some studies that younger age is associated with the recurrence of pressure ulcers (2, 12). One possible explanation is that pressure ulcer patients with SCI were generally much younger than those with other illnesses. Therefore, specific awareness is recommended for young and neurologically disabled patients following surgical treatment of pressure ulcers (5).

DM has been widely recognized as a risk factor for the development of pressure ulcers (38–40). However, in this study, there was no significant difference in the incidence of DM between the patients with and without pressure ulcer recurrence. We believe that the reason is that in the condition of multiple factors contributing to pressure ulcer recurrence, there were potential confounding factors in the analysis. Although individually weighted risk factors based on adequate statistical methods would be useful to assess the role of each risk factor in the development of pressure ulcers (41), this study is limited by its relatively small sample size for doing such work. In contrast, DM has been recognized by decision tree algorithms as a risk factor for the recurrence of pressure ulcers following surgical reconstruction. Machine learning methods may recognize a specific pattern to provide a useful classifier to make predictions for unseen data/objects (42, 43).

This study also demonstrated that patients with recurrent pressure ulcers were significantly younger and had a higher rate of sustaining scoliosis, but a lower rate of Parkinson's disease, than patients without recurrence. It had been reported that pelvic obliquity occurs secondary to scoliosis and results in increased instability of the hip on the high side and ischial decubitus ulcers on the low side (44). In a study of 166 patients who underwent 252 flap procedures, in addition to young age and oblique pelvis, scoliosis was recognized as a factor related to recurrence (45). The observed lower rate of Parkinson's disease in this study seemed to contradict the concept that the prevalence of pressure ulcers was markedly increased when Parkinson's coexisted (46) because the incidence of pressure ulcers is suggested to be inversely related to the amount of movement made during the night. Another large cohort study on more than 87,000 persons with pressure ulcers also revealed that Parkinson's disease was associated with the highest prevalence of pressure ulcers, although this study group did not include those patients following surgical reconstruction. In this study, the decision tree algorithm did not include scoliosis and Parkinson's disease as risk factor classifiers, which may be because of the sacrifice of pruning these relatively small numbers of patients in constructing a decision tree composed of a three-layer structure. Indeed, the reconstruction of more layers in the decision tree model may only increase the fair predictive power in this study (AUC of 0.764), and a decision tree model with too many layers or splits would make the model complex and difficult to use in the clinical setting.

The study was limited to a relatively small sample population to explore a disease influenced by multiple complex factors. Additional limitations of this study should be addressed. The first is selection bias associated with the retrospective study design. Second, socioeconomic factors and other potential factors such as nutritional status, being under- or overweight, anemia, vitamin deficiency, and arterial obstructive diseases were not analyzed or controlled in this study; therefore, some bias may exist. Third, the wound management, rehabilitation process, and activity may differ widely among these patients, which may have led to some bias in the analysis. Fourth, the recurrence of pressure ulcers was limited to those pressure ulcers with stage 3 or 4, because in such circumstances, a surgeon may need to determine whether to perform further reconstruction or allow the wound to heal secondarily. However, if the definition of pressure ulcer includes those with stage 1 and 2 pressure ulcers, the results may be different. Furthermore, the duration of each previous ulcer, the infectious status and pathology of the involved skin region, extension of previous ulcers, the type of spinal cord injury, and the bedridden time of the patients were unknown in this study, resulting in some potential bias in the comparison of the outcome. Whether the study results of bed-ridden patients in this study could be generalized to those who had different ambulatory status require further investigation. In addition, a longer follow-up time of more than three years, as performed in this study, may also impact the analysis of the results. Finally, the study was limited to a single center with a relatively small number of studied patient population, and patient injury characteristics may vary from those observed at other institutions, thereby limiting the generalizability of the findings.

## Conclusions

This study demonstrated that age and SCI were the two most important risk factors associated with recurrent pressure ulcers following surgical reconstruction.

## Data Availability

The raw data supporting the conclusions of this article will be made available by the authors, without undue reservation.
